# Sources of Stress, Family Functioning, and Needs of Families With a Chronic Critically Ill Child: A Qualitative Study

**DOI:** 10.3389/fped.2021.740598

**Published:** 2021-11-04

**Authors:** Chantal Grandjean, Pascale Ullmann, Mark Marston, Marie-Christine Maitre, Marie-Hélène Perez, Anne-Sylvie Ramelet, The OCToPuS Consortium, Anne-Laure Lauria

**Affiliations:** ^1^Institute of Higher Education and Research in Healthcare, Faculty of Biology and Medicine, University of Lausanne, Lausanne, Switzerland; ^2^Pediatric Intensive Care Unit, Department Woman-Mother-Child, Department Woman-Mother-Child, Lausanne, Switzerland; ^3^School of Healthcare, University of Applied Sciences and Arts, Fribourg, Switzerland; ^4^University Children's Hospital Basel, Basel, Switzerland

**Keywords:** chronic disease, chronic critical illness (CCI), pediatric intensive care unit, family, family nursing, family functioning, family stress, family needs

## Abstract

PICU hospitalization is particularly stressful for families. When it is prolonged and the prognostic is uncertain, it can significantly and negatively affect the whole family. To date, little is known on how families with a chronic critically ill (CCI) child are affected. This national study explored the specific PICU-related sources of stress, family functioning and needs of families of CCI patients during a PICU hospitalization. This descriptive qualitative study was conducted in the eight pediatric intensive care units in Switzerland. Thirty-one families with a child meeting the CCI criteria participated in semi-structured interviews. Interviews, including mothers only (*n* = 12), fathers only (*n* = 8), or mother and father dyads (*n* = 11), were conducted in German, French, or English by two trained researchers/clinical nurses specialists. Interviews were recorded, transcribed verbatim, and analyzed using deductive and inductive content analyses. Five overarching themes emerged: (1) high emotional intensity, (2) PICU-related sources of stress, (3) evolving family needs, (4) multi-faceted family functioning, and (5) implemented coping strategies. Our study highlighted the importance of caring for families with CCI children. Parents reported high negative emotional responses that affect their family functioning. Families experience was highly dependent on how HCPs were able to meet the parental needs, provide emotional support, reinforce parental empowerment, and allow high quality of care coordination.

## Introduction

Infants, children and adolescents with repeated or prolonged stays in pediatric intensive care units (PICU) due to pre-existing health conditions have increasingly been reported worldwide. Chronic Critically Ill patients (CCI) are commonly defined with the following criteria: prolonged PICU length of stay or repeated PICU hospitalizations and multisystem disease and/or dependence on technologies to maintain vital functions. Care of CCI patients is challenging due to the complexity of their clinical conditions and the coordination required among caregivers (1–4).

When a child needs PICU hospitalization for an undetermined duration, the entire family unit is affected; the experience being reported as “riding an emotional roller coaster” (5). PICU-related sources of stress include illness severity, prognosis and outcomes, physical appearance and emotional responses of the child, multiple procedures or interventions, and overall PICU's environment (6). Other sources of stress in PICU are related to discontinuity of individualized care (7), under used parental expertise (8), fragmented participation in care and decision-making (9), support of siblings and other family members, organizational and financial burden (10, 11), social isolation (12), and PICU discharge (13). The development of adaptive strategies and the evolution of family needs have also been identified: increasing their medical knowledge, understanding PICU's organization, becoming familiar with the multidisciplinary team, developing family routines in the hospital, adopting new parental roles, and keeping hope alive. Parents of CCI patients specifically ask for clear and coordinate communication, recognition of their expertise and trusting relationships (14). During hospitalization, family functioning can be disrupted. In this context, family functioning is defined as family members' ability to preserve interrelated relationships, perform family roles while adapting to new roles and routines, cope with family problems, and communicate effectively (15). A lack of cohesion, support or expressiveness between family members, and family conflicts, impact negatively not only on the child's physical and psychosocial health but also on quality of life (16, 17).

In order to prevent or minimize negative psychological outcomes and better target family needs of CCI patients, it is important to identify the preventable sources of stress, the family functioning challenges and needs that are specific to this population throughout the PICU stay. The aim of this study was to explore sources of stress, family functioning and needs of families of CCI patients, specifically during PICU hospitalization.

## Materials and Methods

### Design

**Figure 1:**
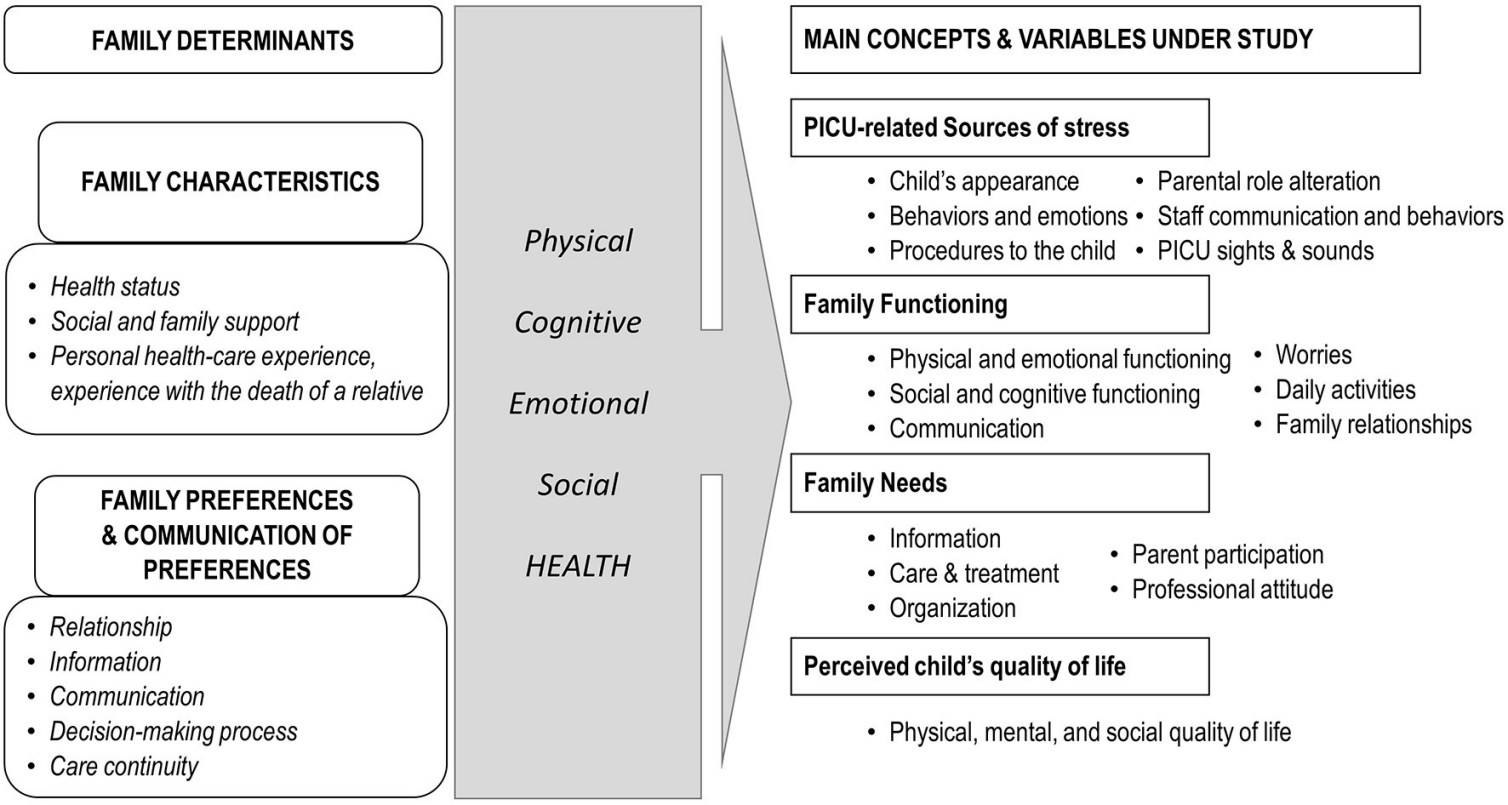
Theoretical model of the study. Illustration of links between the family determinants (4, 22), the domains of family health (23) and the concepts and variables under study (24–27).

This study is part of a larger research project entitled “Measurement of the impact of CCI hospitalization on families over time: the OCToPuS 2 study” which has a qualitative analysis inside a larger quantitative study. We used a triangulation design with a convergence mixed-methods model in which qualitative and quantitative data are collected and analyzed separately before being compared and contrasted in final interpretation (18). With such design, results can be reported in separate papers (18). Therefore, here we report the qualitative component only. For this qualitative part, a descriptive qualitative design was used (19). We opted for qualitative description, because it allows to stay close to the data and to have little inference in the interpretation process (20). This is particularly relevant for later interpretation when both quantitative and qualitative results are compared and contrasted. The philosophical assumptions of OCToPuS 2 study are anchored within the interpretative framework of pragmatism (21). A theoretical framework described in Figure 1 guided the entire research process, including the development of the interview guide and the data analysis. It illustrates how family determinants, family characteristics and preferences, including the way they are communicated, may affect the domains of care (4, 22). In order to explore the impact of CCI hospitalizations on families, family characteristics and preferences were linked with the four domains of family health (physical, cognitive, emotional, and social health) of the Post-Intensive Care Syndrome in children (PICS-p Framework) (23). These domains are not only central, but also interrelated, in the same way the child's and the family health influenced each other. In our study, family health domains included PICU-related sources of stress (24), family functioning (25), family needs (26), and family assessment of their child's quality of life (27).

### Setting

This study was led in the eight accredited level II & III PICUs in Switzerland, which count 100 beds and ~4,000 annual admissions. They admit a broad case-mix of medical and surgical patients, including neonates. Six units were located in the German-speaking and two in French-speaking parts of Switzerland. Current PICU staffing practices include 8- or 12-h shifts. Family visiting practice was similar across all participating units with no restrictions, except since March 2020 until the end of data collection due to the COVID-19 outbreak, when only one family member was allowed at the bedside.

### Population

Family members of CCI patients hospitalized in PICU who participated in the larger OCToPuS 2 research project were eligible for this qualitative part of the study. The operational definition of CCI patients for this study was infants, children and adolescents (age range: 1 month to 18 years) who were technology dependent AND hospitalized in a PICU for a duration of ≥8 days (post-term corrected age for former pre-mature infants) or those who had ≥2 PICU admissions in the last 12 months (1, 2, 4). Dyads of family members were sought out when possible. Family members could include mothers, fathers and significant others whether they were blood-related or not to the CCI patient (e.g., siblings, grandparents, careers) (28). Family members had to be fluent in French, German or English. Family members of CCI patients were excluded if there was a possibility of withholding or withdrawing life-sustaining medical treatment, as outside the scope of this study.

### Sampling and Recruitment

To allow maximal diversity and representativeness of families' experiences and perspectives, a purposive sampling technique was used. Maximum variation was also sought with respect to different CCI conditions and PICU characteristics (29). Sample size was determined to recruit ~10% of the total (*N* = 247) consenting families of the quantitative part of the OCToPuS 2 project. Recruitment was performed by the local investigators in each of the eight PICUs until this target sample size was reached. Two research collaborators contacted the families who consented to this qualitative part of the study by telephone to arrange for an interview within 2 weeks of their child PICU discharge.

### Data Collection

**Figure 2:**
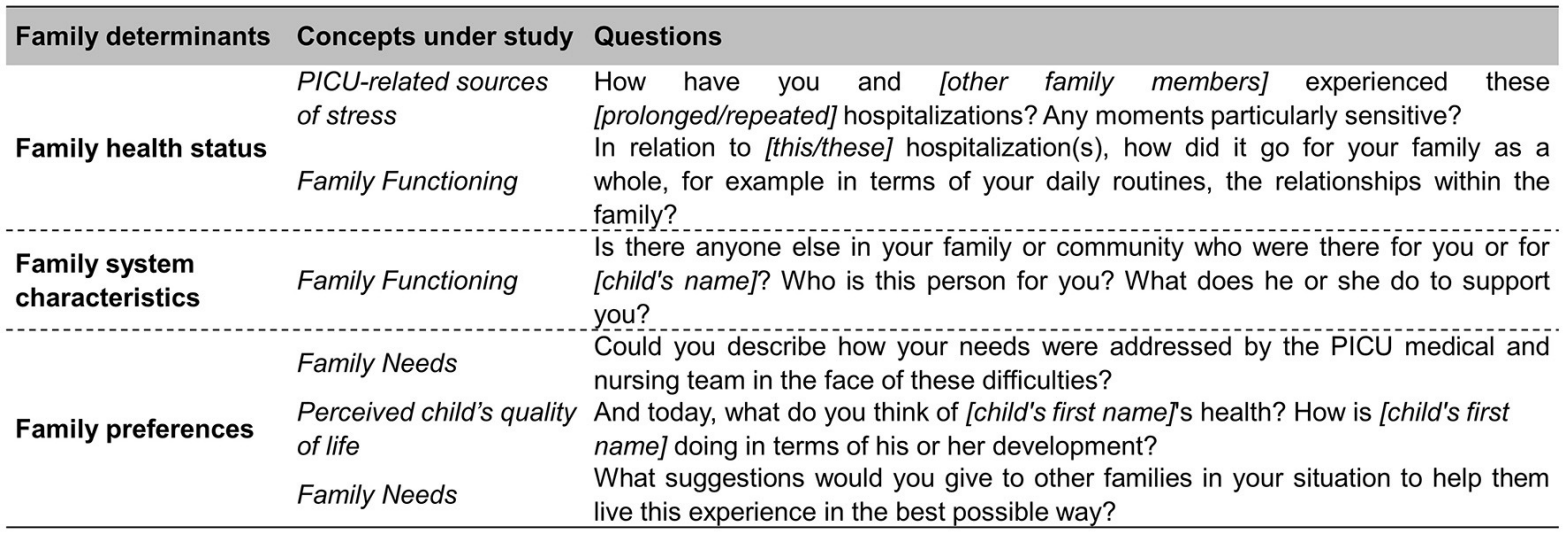
Illustration of the development of the interview guide based on the family determinants (4, 22) and the concepts under study.

Following ethics approval, data were collected using semi-structured interviews, between November 2019 and August 2020, including a 3-month study suspension period due to COVID-19 health restrictions. In cases where two family members of one child consented to participate, multifamily members' interviews were held. Two collaborators of the research team (CG, MM) conducted the interviews; both are advanced practice nurses who completed a master's degree program, with a former pediatric intensive care specialization diploma and more than 5 years of experience working in a PICU. None of them were involved in direct patient care and had previous contacts with the participants. Both were also trained in qualitative research methods. The interview guide was developed based on the theoretical framework of the study described in Figure 1. In order to explore CCI hospitalizations' impact on families, the four main concepts under study (PICU-related sources of stress, family functioning, family needs and perceived child's quality of life) were used to build the main questions of the interview guide. To illustrate this process, we provided an example of how the model guided the formulation of questions in Figure 2. Before the start of data collection, two pilot interviews were conducted with a parent who did not take part in the study; this enabled refinement of the interview guide and improved interview skills. Each interview started with a short introduction followed by the opening question “*Could you tell me why your child was admitted to the intensive care unit and the reason for his or her [prolonged/repeated] hospitalization?”. One* final question was asked at the end of the interview “*What suggestions would you give to other families in your situation to help them live this experience in the best possible way?”*. Interviews were conducted within 15 days after PICU discharge at a location selected by each participant, either at their home, in a private room at the hospital or by secured online video conferencing (due to COVID-19 restrictions). The interviews were audio recorded and transcribed verbatim by French, German and English-speaking professional transcribers. All identifying information was anonymized. It was possible that interviews could bring up unexpected negative emotions or distress in participants. In that case, participants were offered psychological support available in the study settings.

### Data Analysis

All transcribed interview data were analyzed using the content analysis method described by Mayring (30) and a qualitative data analysis software (MaxQDA 2020 Analytics Pro 2020 version 20.0.8) (31). The adoption a qualitative content analyses method was justified by its coherence with the qualitative description design used in this study (32). A systematic approach to deductive and inductive coding and categorizing textual information following standardized steps allowed a rigorous method of data analysis. Prior to data analyses, the main analysts (CG and PU) reviewed and acknowledged their own biases and pre-conceptions. First, a coding scheme was constructed by both analysts based on the study's theoretical model (Figure 1). This construction was closely connected with the four main family health domains under study (PICU-related sources of stress, family functioning, family needs and perceived child's quality of life). In this way, the deductive approach of the qualitative content analysis was respected, while at the same time allowing for an inductive approach. Then, a pilot phase to test the coding guide was performed with the three first French interviews, allowing the development of preliminary coding and the addition of coding rules. In the next step, an immersive reading and coding of each interview was done by the research collaborators individually according to the language used in the interviews. Data saturation was achieved after conducting and analyzing 31 interviews (33). In the end, the list of codes were analyzed to determine how each code fit into an overarching theme. Each theme was identified and analyzed in relation to the aim of the study. Analysis of each family unit was performed for each interview, regardless of the number of family members interviewed. Credibility of analyses was ensured by the number of interviews in three different languages, the involvement of parents across seven of the eight different study sites, and the use of three different persons to collect and analyze the data.

## Results

Sixty-eight families were eligible/agreed to participate and 31 (46%) participated to the study. Reasons not to participate were: lost during covid-related study suspension period (*n* = 11, 42%), no longer reachable (*n* = 10, 39%) and withdrawal (*n* = 5, 19%). Thirty-one interviews were conducted in total, including 20 interviews with one parent and 11 with two parents (*n* = 22). The participants came from seven of the eight study sites involved. Interviews were conducted in German (*n* = 19, 61%), in French (*n* = 10, 32%) and in English (*n* = 2, 7%). Sixteen (52%) interviews were conducted face-to-face and 15 (48%) interviews were carried out *via* online videoconferencing. The interviews duration varied from 40 to 80 min. Table 1 summarizes the demographic characteristics of the family members and their child. Participants included 23 mothers (55%) and 19 fathers (45%) of CCI patients. More than half of them were Swiss (62%, *n* = 26) and were educated at tertiary level (59%, *n* = 24).

**Table 1:** Demographic characteristics of the sample of participants and their CCI children.

**Number of interviews with one participant (*N* = 20), *n* (%)**	**20 (65)**
**Relationship to the CCI child**, ***n*** **(%)**	
Mother	12 (60)
Father	8 (40)
**Nationality (*****n*** **=** 19)^a^, ***n*** **(%)**	
Swiss	11 (58)
European	8 (42)
**Education (*****n*** **=** 19)^a^, ***n*** **(%)**	
Tertiary	9 (48)
Secondary	9 (48)
Compulsory school	1 (5)
**Number of interviews with dyads of two participants**	**11 (35)**
**(*****N*** **=** **22)**, ***n*** **(%)**	
**Relationship to the CCI child**, ***n*** **(%)**	
Mother	11 (50)
Father	11 (50)
**Nationality**, ***n*** **(%)**	
Swiss	15 (68)
European	5 (23)
Non-European	2 (9)
**Education**, ***n*** **(%)**	
Tertiary	15 (68)
Secondary	7 (32)
**Type of CCI patients (*****N*** **=** **31)**, ***n*** **(%)**	
With prolonged PICU hospitalization	23 (75)
With repeated PICU hospitalizations	8 (25)
PICU length of stay, (Mdn, IQR)	17 (9–29)
**Age (*****n*** **=** **30)**^a^, ***n*** **(%)**	
1 to ≤ 12 months	15 (50)
≥1 to ≤ 5 years	4 (14)
≥5 to 18 years	11 (36)
**Diagnostic group (*****n*** **=** **30)**^a^, ***n*** **(%)**	
Digestive	8 (26)
Cardiovascular	6 (19)
Respiratory	4 (13)
Neurology	4 (13)
Oncology	4 (13)
Other	5 (15)

*CCI, Chronic Critically Ill*.

a *Missing data*.

### Qualitative Themes

Supported by the study's conceptual model (4), deductive content analyses resulted in four central themes that characterize the specific PICU-related sources of stress, family functioning and needs emerging in families of CCI patients during a PICU hospitalization, including as follows: (1) high emotional intensity, (2) PICU-related sources of stress, (3) evolving family needs, (4) multi-faceted family functioning. From inductive content analyses, one theme emerged (5) implemented coping strategies. Table 2 describes the five central themes with 18 categories and 46 subcategories.

**Table 2:** Themes, categories and sub-categories resulting from data analyses.

**Categories**	**Sub-categories**	
**Theme 1. High emotional intensity**		
Negative emotions	• Shock • Fear • Stress	•Anger •Guilt •Sadness
Positive emotions	•Relief
	•Gratefulness
**Theme 2. PICU-related sources of stress**	
Uncertainty in Health Outcomes	•Severe diagnosis and uncertain prognosis
	•Repeated procedures, equipment and potential complications
	•Child's appearance and behavior
Care environment	•PICU physical environment
	•Nurse ratio
Alteration in parental role	•Presence and protection
	•Parental expertise
	•New parental roles
**Theme 3. Evolving family needs**	
Relationships with the multidisciplinary team members	•Trust
	•Support
Communication	•Types of communication
	•Content
	•Preparation
Involvement	•Presence at the bedside
	•Care participation
	•Decision-making
Care coordination	•Multidisciplinary team
	•Continuity
	•Individualized care
PICU discharge	•Transition
	•Preparation
**Theme 4. Multi-faceted family functioning**	
Living in Hospital	•Accommodation
	•Daily life routines
	•Financial burden
Role of relatives and the parents as a couple	•Emotional and organizational support
	•Social isolation
	•Complementarity of parental roles
Siblings	•Separation
	•Impact
	•Integration
**Theme 5. Implemented coping strategies**	
Physical health	•Maintain daily activities
Emotional health	•Develop resilience
	•Find the best strategies to deal with own emotions
Social health	•Take opportunities to have a break
Empowerment	•Trust in the PICU team
	•Facilitate good communication with HCPs
Future as a Family	•Unknown future
	•Back to normality (as much as possible)

### Theme 1: High Emotional Intensity

Participants experienced various emotions that were particularly difficult and high in intensity. They expressed feeling *Shock, Fear, Stress, Anger, Guilt*, and *Sadness*. They also reported positive emotions of *Relief* and *Gratitude*. Particularly at the time of the PICU admission and diagnosis, participants described being in a state of shock that felt like being in an unreal world, “*in another world*” or “*in a sitcom*,” or a “*blackout*”:

“*[…] I still remember the room and the people I was talking to, but for the remainder of the meeting […] I was inactive […] I was in my thoughts, I was in a void […] I wasn*'*t thinking about anything, I was almost transparent […].”* (Father, interview 6)

At the beginning of hospitalization, fear and stress were related to the uncertainty of child's vital prognosis, surgery, medical procedures, and complications. While the illness was progressing, fear and stress remained present, but were increasingly related to the child's long-term outcomes and future:

“*[…] what seems to be the most relevant step back is she lost the ability to swallow […] Swallowing whilst breathing wasn*'*t possible at all. She still doesn*'*t really do it. And that is the biggest – the biggest problem that we have just now. […] she would like to eat, because she can remember how it went. But her body can*'*t cope. And that is a huge frustration to her.”* (Mother interview 24)

Anger was expressed when participants felt disrespected in their parental role by healthcare professionals (HCPs), while guilt and sadness were emotions related to feeling that they had '*done wrong'* as parents:

“*[…] It feels difficult to me to assume the fact that her scar can be seen and to have to explain what has happened […] and as a matter of fact, even toward our neighbors who… well, who know M. since he was a baby […] I tend to avoid them because I don*'*t want to talk about it […] all discussions are considerate, but… I don*'*t feel comfortable with it.”* (Mother interview 19)

Positive emotions, such as relief, joy, gratefulness, were mainly reported after successful medical procedures and occasionally at the PICU discharge:

“*[…] After the surgeon called and told us the operation had gone well, […] I was relieved because M. was alive. So when I got to the PICU I didn't see all the equipment around at all, I just saw M. who was alive.”* (Mother interview 19)

### Theme 2: PICU-Related Sources of Stress

Participants experienced varying intensities of stress due to numerous PICU-related factors. Three main categories of PICU-related sources of stress were: *Uncertainty in health outcomes, Care environment* and *Alteration of parental role*.

#### Uncertainty in Health Outcomes

First, uncertainties were related to the prognosis of the severe conditions, multiple medical procedures, real or potential complications, and medical errors:

“*[...] I find that, this was difficult to live with, because we had the impression that we were making progress, once he had extubated him and that. after that weekend when he had these epileptic seizures where there had been this famous gesture [...] this error, then on Monday morning they told us: ‘Well, now we're going to have to reintubate him'. pff. it was. it was complicated. [...].”* (Father interview 19)

As the illness progressed, participants became concerned about the change from an acute to a chronic health condition with its related ups and downs:

“*I think he, I mean, he's otherwise not having luckily other problems besides his digestion. I mean, it could still have been worse if he, I mean, there are conditions that really impair you from doing, like, normal things. And I think that one day, hopefully, I think he can do normal things, but we don't know how he will be able to eat while he*'*s growing. There is still a lot of hope that he will get better […].”* (Father interview 4)

Physical appearance and psychological behavior changes of their child were other important sources of stress for participants: equipment deforming the child's body, agitation and cries when pain and sedation were poorly managed, and the limitations caused by long-term equipment and medication:

“*For N. it was really very very difficult because he is a boy who loves to talk so of course he couldn't talk. So. He wrote on a writing pad but as he had morphine [...] he wrote very badly, we didn't understand, so he got so angry, I've never seen my son like that, he was so angry that sometimes he threw the pen there, he threw it at me, at the nurses so much that he was. Ah really those were moments that were. very difficult.”* (Mother interview 18)

#### Care Environment

PICU units were described as intimidating and gloomy: “*it's another world*,” “*it lacked color, joy of living*.” The current PICUs environmental design highlighted the lack of privacy and confidentiality needed for participants, particularly in the waiting room. They noted that the lack of light and room, and constant noise resulted in their children's loss of time and space:

“*[…] in the evening, there was a lot of noise, even from the medical staff, [...] it was a disaster, it was a real henhouse and it's true that it got on our nerves a bit. It wasn't every night, [...] it was with a couple of persons, [...] but at the time it annoyed us.”* (Mother interview 9)

The participants experienced stress due to the noise caused by too many visitors, fear that another PICU patient could be contagious to their own vulnerable CCI child, and upset to be confronted with End-of-Life situations:

“*It was tough for me to hear the penetrating cries of a mother from the next-door room [...] it's such a screaming and crying at a very, very. I can't describe it [...] and I had a bit to nibble on, but never mind [...] I also understand the parents and they should be crying. That is important too. They are allowed to, they have to.”* (Mother interview 11)

However, meeting other parents was also reported a source of comfort and support:

“*What we forgot, that also helps too, other than doctors, the medical teams and people around us, is to talk to other parents too [...] we talk about our children, what's wrong with them, what's the next step, etc. It may be people we will never see again, but for a week, two weeks, a month, we form a little team in the waiting room [...].”* (Father interview 6)

As recovery progressed, children required less complex care and decreased nurse-to-patient ratio; this caused significant parental stress, especially when participants were not allowed to stay at the bedside, as much as they would have liked:

“*So at the beginning [...] there was probably the most experienced nurse in the whole staff looking after him and probably two other standing around and helping her. And then, you know, when he got better, it felt like [...] the less experienced nurses or the training ones. And at the beginning, that worried me a little, when [...] you've come from like the super nurse to like the normal good nurses.”* (Mother interview 15)

#### Alteration in Parental Role

Participants tried to maintain as much as possible their presence at the bedside of their child, so that he would never be alone.

“*For us, the need really was that actually someone always stays with him, with occasional exceptions. But, in this way, we didn't want - especially when he was having the pain-episodes, that all of a sudden he has this pain and he's just alone.” (Mother interview 8)*

To maintain their protective role, participants were intense advocates for their children, particularly in cases of repeated PICU hospitalizations. Their parental expertise was claimed as being a skilled caregiver at home or already having experienced several hospital stays:

“*I told our primary nurse, after my last stay in hospital, after we had adjusted our care plan once again, that this wasn*'*t possible. So slowly I learnt to express myself and to say: Until then and no further.”* (Mother interview 7)

The absence of consideration for parental expertise was often experienced when HCPs did not listen to or involve participants in decision-making.

“*There was a situation where I just didn't feel taken seriously, with the expertise that I have. And afterwards we had another unfortunate conversation with the pneumologist, who then got involved, but somehow not officially and too late. And that was something, I'd say, internal to the departmental, across disciplines, problem that we got to feel later on.”* (Mother interview 8)

First-time parents needed to adjust to their new parenting role, because it was not what they expected. Parents with a child whose conditions was becoming chronic had to learn a new caregiver's role:

“*[…] I just said today, in care, changing the collar of the cannula, I remember, at the very beginning this was the highlight of our day, when I went with my husband, we, well I, was nervous in the morning because we had to do it today. And now, after this month at home, during which we did it every day, it's like brushing our teeth.”* (Mother interview 31)

### Theme 3: Evolving Family Needs

Family needs described by the participants evolved over time and throughout their child's hospitalization. They were related to the *Relationships with the multidisciplinary team, Communication, Involvement, Care coordination*, and *PICU discharge*.

#### Relationships With the Multidisciplinary Team Members

For the participants, strong relationships with HCPs took place when trust was possible and adequate support provided. The professional skills that help to build and maintain this trust included professionalism, compassion, calmness, and transparent communication from the whole team and throughout the PICU stay:

“*It was a good atmosphere with the nurses, the (care) assistants too, who should not be forgotten because they often come to help. It was a bit like the sunshine of the day, seriously […] they don't do the care, they help out with washing, and there's another dimension to the nurse […] it was a little bit the leisurely side […].”* (Mother interview 9)

Participants reported the benefits of living in a country with efficient healthcare services, in particular for their child's chances of survival and for having highly trained HCPs. However, they also noted the downside of being in a university hospital, in which there is a significant proportion of staff in training. Variations in clinical care practice and expertise between the different HCPs contributed to parental frustration:

“*Sometimes there are nurses, that have been there for 20 years, they change infusions [...] they are precise, it doesn't shake and then the next day there is a young [nurse], she has to learn [...] It's a university hospital so [...] we are aware of that, but when it's your child who is already in a critical situation, it's complicated [...].”* (Mother interview 19)

#### Communication

High quality of communication came up frequently as a way to consolidate the relationship of trust. Participants needed time, anticipation and repetition of clear and transparent information, to ensure they were understood. The language barrier appeared to be an additional challenge for non-native speakers, although for some, not understanding everything was perceived as a way to protect themselves from stress or anxiety. Having to actively seek information was a stressor that did not meet their needs:

“*I thought this could be optimized, that […] you don't have to ask for it, that it's clear that every week, you make a small meeting in a room […] and you discuss the case. What happened last week? What is going to happen next week? So […], you're on a track.”* (Father interview 4)

Participants valued information that was tailored to their needs. During their PICU stay, participants were able to identify the right person for the right information:

“*The daily program for example, well we ask the nurses, if it's about values, if it's about projects or if it's about questions concerning an operation, is it today, is it tomorrow, [...] well it'll rather be the doctors.”* (Mother interview 9)

Many participants suffered from a lack of preparation, regarding their child's physical transformation and/or the PICU environment, but they felt better prepared regarding the eventuality of a complex care trajectory and treatment plan during the PICU stay:

“*[…] we met Dr. K. and he explained the situation to us. We were […] in this little room near the intensive care unit. He did a little presentation and he was already talking about different things. That was: how long were we going to stay here. That was very good because even though it might be a bit stressful. But you then know for how long we would stay here […] and he said one month.” (Mother interview 1)*

#### Involvement

Participants described how they became involved in care and decision-making about their child throughout the PICU stay. For some, simply being able to be present at their child's bedside was enough to make them feel welcome; fear of the equipment and pain were the main reported reasons for not actively participate in care. For others, it was essential to feel helpful and offer spontaneously their help for hygiene care, massage, mobilization or pain management for instance. Participants also reported variable support from HCPs in the facilitation of their participation in care. In situations where technology-dependent children were ready to be discharged home, parental preparation was perceived crucial by HCPs, and parental participation in care was formally sought as a result:

“*We both had to learn it, we sometimes had to go there together, because you have to change the cannula and the collar and so on, we [...] usually are together two or three times a week, at one point my husband is alone and the rest [of the time] am alone.”* (Mother interview 31)

Regarding parental participation in decision-making, variable involvement was reported. Some participants expressed having sufficient trust in the HCPs' competencies to let them make decisions about their child's care. For others, mostly participants with repeated PICU hospitalizations, a full understanding of their child's health condition, was essential to be able to actively participate in decision-making. In all cases, participants needed to be asked to what extend they wished to participate in care and decision-making:

“*[...] this tube in his nose [gastric tube] was bothering him [...] and she [the doctor] said: ‘For me if you think he's feeding normally, if you want, we'll take it out. How do you assess it?' and we said: ‘But can we decide?' and she said: ‘Well, if you feel like it, yes.' So we took it off and M. started to eat.”* (Mother interview 19)

#### Care Coordination

Participants reported up to 15 different professional disciplines involved in their child's care both during PICU hospitalization and directly after discharge. Social support was proposed for the physical, psychological and spiritual health of the whole family, as well as for administrative and financial needs related to their child's hospitalization. Due in part to the large network of different professionals involved, participants reported a lack of care continuity affecting follow-up, including transmission of information about treatment plans, decision-making processes and consideration of children's specific needs. Unfamiliar staff resulted in the lack of trust, especially when participants had to repeat information over and over again:

“*[...] I have to tell the same things over and over again and [...] but mistakes also happen because they don't read the documentation properly or the communication isn*'*t correct and I think that could easily be prevented if they were more or less always the same.”* (Mother interview 31)

Having a primary nurse or physician appeared to be a way to overcome the lack of continuity:

“*[…] he [the chief surgeon] has been the number one person of reference for us. Even though there have sometimes been situations where he himself did not want to be in this role - nor could he be, given the possibilities. But we had complete trust in him. Because the other staff rotated so much - purely in terms of shifts - that you have some reference person somewhere on paper, but [...] you haven't seen them for weeks at a time.”* (Father interview 25)

Consideration of specific needs appeared to be a fundamental element in supporting the child's recovery process. Special attention to meet the individual needs of the child included single room, regular space-time reference points, protected circadian rhythm, dedicated play area in the unit, personalized book with pictures of the child, tablet to communicate despite the equipment, birthday party, visit from the family pet, and week-end at home:

“*When she had her tracheotomy. [...] she was depressed she was always crying looking at the picture of her dog. [...] So they allowed us to take her dog [...] We didn't take it to the intensive care unit but we went to the back yard. [...] a week later, she no longer had oxygen, she was back in her wheelchair and she came several times in her wheelchair to see her dog.”* (Mother interview 2)

#### PICU Discharge

Participants described the PICU discharge as a moment of relief and joy:

“*[...] it was a sign to me, now he is no longer, well, now he can no longer suddenly die. […] And then I could start allowing feelings. […] I went to visit him without feelings and without mother's instinct. And that's when [...] I could start admitting feelings. That was kind of the decisive point for me.”* (Mother interview 27)

However, participants also expressed fear of leaving a familiar and safe place:

“*When we came up here [to the ward], some nurses didn't even know what a drainage really is – well, how it works. And that - when we saw that, we also thought: Yes, are we in the right place? Because downstairs [in the PICU] they knew exactly how to handle these things, how to deal with it, […] how to move it, how to hold it so that nothing happens. Because these are quite particular. It's not something you see every day.”* (Mother interview 13)

The preparation for PICU discharge seemed to vary greatly according to the experiences reported. Most participants described a rapid, unanticipated and unprepared discharge, resulting in a sudden increase in the organizational burden and stress:

“*I don't mind being there [in the ward] permanently, but I do mind not being told up-front that […] somebody possibly assume you will be suddenly able to take full-time care of a child, which before has been in PICU all the time.[…] I sat there, and I'm, like, in this room by myself, with my son in the bed. And I'm like, ‘Okay, so now I'm supposed to change him.' I don't know how to do that. I'm supposed to, whatever, make him eat, drink, what else is expected of me?”* (Mother interview 15)

Some participants highlighted the importance of being prepared with effective care coordination after discharge with for instance a visit to the transfer unit or involvement of the palliative care team:

“*[…] we got intensive first aid courses directly from the PICU, from the senior doctor [of the palliative team] - just really for me and my wife - specifically tailored to us, tailored to our son, with a catalog: “How do you put in an intestinal tube?” “How do you put in a feeding tube?” All eventualities that could occur or could have occurred were shown to us and also given to us in the form of, yes, information sheets.”* (Father interview 21)

### Theme 4: Multi-Faceted Family Functioning

In the context of our study, family functioning was represented through five themes covering different dimensions of family life: *living in hospital, roles of relatives and the parents as a couple, siblings*.

#### Living in Hospital

Access to hospital accommodation for participants was a fundamental response to the need for proximity:

“*we could go and have a rest, we could sleep at night knowing that if we got a call we were there within two minutes. and that's great.”* (Father interview 19)

At the same time, participants had to accommodate to COVID-19-related restrictions and take turns to be present at their child's bedside. For the participants, such parental visit rotation had the advantage of assuring a constant presence at the child' bedside, and facilitating learning and communication with HCPs. However, the disadvantage was feeling of being more isolated and decreased communication between parents.

“*[...] because of the Covid - Coronavirus [...] we very rarely were together. And if together, maybe for half an hour or so. But in fact the impression of intensive care is that we are separated from each other [...] because we were always alone. But then we exchanged information via our smartphones […].”* (Father interview 22)

Families have also created daily routines in hospital, for example mealtimes together with food brought from home, presence and visits with siblings, sleeping at home:

“*As from the beginning on, in a way, we also introduced rituals there. [...] During the day we were in the hospital and in the evening, when one was back home, there was the normal evening rhythm. […] So what we always did, before we went to bed, we called the PICU and asked how he was doing, what was going on [...]. And so we went to sleep and that was enough for us.”* (Mother interview 20)

Finally, although most participants benefited from paid parental sick leave, some expressed some *financial burden due to their child's prolonged hospitalization:*

“*[...] I used to be self-employed. so I used to work just enough on certain days to be able to come and take care of my child in hospital. Now I'm working for someone, because well it all went down the drain. financially it's crap [...] there are bills that haven't been paid because. I considered that being with my child came first.”* (Father interview 5)

#### Roles of Relatives and the Parents as a Couple

The participants described extensive reorganization required by their child's hospitalization:

“*[...] He [the hospitalized child] will probably be allowed to come home by the day, at weeks. And just go on dialysis at night. Now we are seriousely organizing transportation: Parents-in-law will drive, godfather will come and pick-up at sometime [the older brother]. So. Yes, organisationaly speaking it's a challenge, absolutely. But thank God I have an employer who has extremely family-friendly views, who says ‘family first' [...].”* (Father interview 26)

Mostly parents, siblings and neighbors of participants were present to provide emotional and organizational support. The presence of friends was appreciated by participants, because it created an opportunity for them to think about and do things unrelated to their child's illness:

“*Sometimes it started [...] by giving news, explaining how the day had gone [...] and then afterwards, I believe it was more to change my mind and then. and kind of talk about something else. by talking stupid stuff. [...].”* (Father interview 19)

Although the support of relatives was reported as fundamental, it was also perceived as a burden, due to inadequate relationships or inappropriate request of information. This later led participants to limit their social interactions with relatives and friends:

“*[…] people don't quite realize, to be totally honest and I don't have the energy to always have to explain all that is going on. It's also to protect the family members because well, in intensive care when you arrive it's quite impressive too so. there was a little bit of both, but above all to protect us [...].”* (Mother interview 19)

Participants highlighted the problem of their child's social isolation when hospitalized, especially adolescents who needed to maintain social relationships with friends and keep track with their studies. The importance of being able to dialogue as a couple about one's own emotional state and exchange on the child's treatment plan was highlighted:

“*[...] it's my husband who straightened it out to me because he used to say: ‘Oh, it's pipes, it can be repaired, it's not much'. [...] He was always sure that it was going to work [...].”* (Mother interview 16)

#### Siblings

Separation between parents and siblings, or between sibling members, brought up a sense of guilt to the participants, while the need for participants to be fully available to their hospitalized child predominated. Siblings' visiting restrictions due to the COVID-19 pandemic context had a negative impact on the possibility of the whole family being together. Main impact on siblings, as perceived by the participants, was related to intense feelings of fear and misunderstanding, manifested for example by bed-wetting or high irritability. The participants also described their efforts to support the siblings by providing them adapted information:

“*But we are always trying to say [to the siblings]: the hospital is not something bad in some way. Of course, we have experienced something bad. But now it's also about helping S. again and then letting her get healthy. [...] That's what you have to discuss with the children always and again and also explain and prepare them for when we leave and how it will be during that time, who will be there, who will look after them.”* (Mother interview 12)

### Theme 5: Implemented Coping Strategies

Participants named a list of coping strategies they had implemented during their child's hospitalization at PICU. These strategies were related to the promotion of their *physical, psychological* and *social health*, the development of parental *empowerment*, and the *future as a family*.

#### Physical Health

Our results indicate that maintaining good physical health required attention to maintain daily activities as much as possible, including nutrition and hydration, bathing/showering, getting a minimum period of sleep, and going for a walk or doing some sport:

“*[...] because we would like to stay with our child, we would like to hold his hand all night long, but we have to push ourselves to see the need to rest for him so that the next day we can be for him again because over the long term. we couldn't stand it, and then eat and drink, that's. important, and then we also see something else because all the time in intensive care it's impossible to go through all that.”* (Mother interview 9)

#### Psychological Health

Participants considered they choose the type of psychological support from professionals according to their own needs, including psychotherapy during and after the PICU hospitalization, or other complementary therapies such as hypnosis, magnetizer, acupuncture. Parents named numerous types of coping strategies, including positive thinking, being patient, letting go, avoiding mental scenarios, setting deadlines or goals, being with “what it is,” moving forward one step at a time, nurturing hope and faith:

“*[…] to remain positive (smile). positive and patient and then to trust […] we can let go because there's nothing we can do, apart from being there, so. that's it, and then to take things as they are, and not to want to do things differently or for things to be different.”* (Mother interview 16)

“*[…] I write like a diary for R. I started writing these points down in my iPhone notes, because I lost track of them relatively quickly. After three or four days, my head was so full that I had to write it down. And then I also started taking photos of her. So every day at the PICU, when I came in the morning, I took a picture of her. One close-up and one of her, of her whole body. So that I would have a reference value for myself, so to speak, now also for the days that came afterwards and also for her.”* (Mother interview 24)

#### Social Health

Regarding their social life, the participating families tended toward normality to protect the family system, by spending days at home as a couple or with siblings. Doing activities with friends was also a strategy. For participants of children with pre-existing health conditions, PICU hospitalization offered them an opportunity to take a break from their caregiver's duty of care:

“*[About her periods of absence] I think at first he didn't realize - he was half out of it, but then he knew that I was always reachable, so he knew he could call me, [...] I think it was OK, and he's a very intelligent boy, and then he must have realized that I was fed up and couldn't take it anymore.”* (Mother interview 18)

#### Empowerment

Developing trust enabled participants to actively request information, dare to ask questions, and claim their rights to get answers. Some participants prepared their questions before the meetings with the multidisciplinary team, or determined in advance modalities for transmission of information. Facilitating good communication with HCPs meant daring to communicate their feelings, what they liked or disliked, and avoiding the things that were not said. Actively asking for administrative and financial support was also a strategy:

“*I don't mind asking questions, saying ‘I don't agree with this, but why are you putting this in place?' I think you have to ask all the questions, all the apprehensions that the parents may have because the child feels everything, so if a parent can see that he or she is a little bit pensive or the child is making a whole film, so. Because he has a lot of imagination, so the best thing is to talk about it.”* (Mother interview 9)

#### Future as a Family

Participants spontaneously mentioned uncertainties and fear about the long-term health outcomes of their child, the impact it would have on family functioning, and the necessity to adapt to a new daily life. However, most of the participants also recognized being able to adapt to their new reality:

“*[...] they told us, you can do everything (laughs) with cannulas, you can travel, you can go up mountains and have a look [...] I already have so much more confidence in my abilities to deal with a child with a cannula that I, I think, if it were summer now, I would already go to the swimming pool with him, although I would have to take the ventilator with me and the emergency bag.”* (Father interview 31)

## Discussion

This national qualitative study is the first, to the best of our knowledge, to report CCI family experiences with a focus not only on stress and unmet needs, but also on family functioning. It was guided by the model of interactions between families, clinicians, and ICU determinants influencing goal-directed care for infants and children with chronic critical illness (4). The model provided a theoretical base to guide the development of the interview questions. Although it focuses on goal-directed care of the child, it was useful because it also takes family preferences and family communication of preferences into account. Based on our results, this model could include the coping strategies of families as an important factor impacting their participation in their child's care and decision-making abilities. Our results show that CCI patients' families are affected in various ways throughout the PICU stay that is either prolonged or recurrent. On one hand, they experienced high intensity emotions due to stressors related to the PICU environment and their specific child's complex health conditions. Their needs evolved over time throughout their child's illness trajectory. Therefore, their family functioning was affected in all dimensions. On the other hand, this study demonstrated the abilities of families' adaptation by developing coping strategies over time.

**Figure 3:**
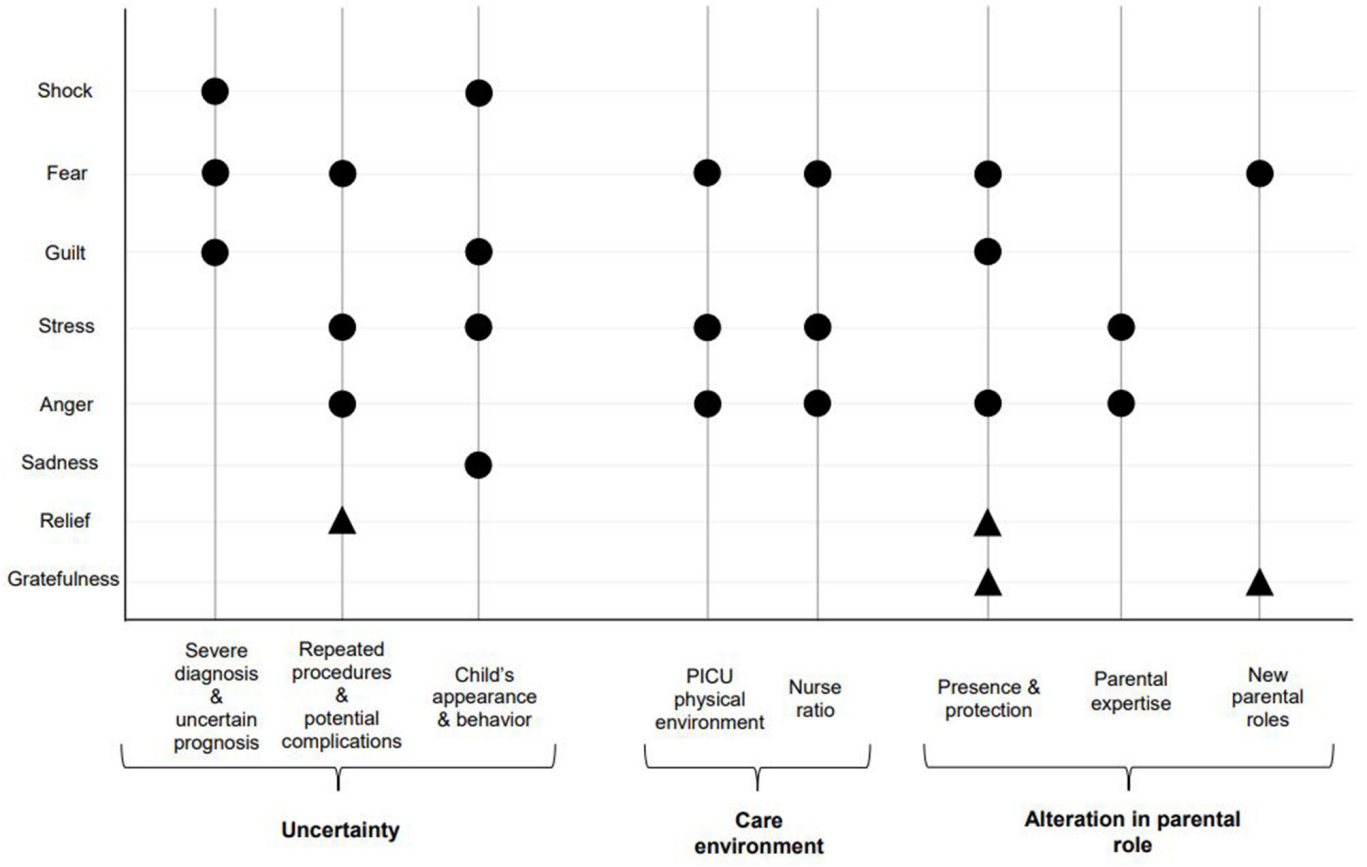
Families-reported emotions in response to PICU-related sources of stress, throughout the PICU stay. ∙: Negative emotions ▴: Positive emotions.

Our results show participants experienced a large array of negative emotions related to stress in PICU, similar to what has been described as being on an emotional roller coaster throughout the PICU stay (5, 6). Reported emotions, such as fear and sadness, can when prolonged lead to anxiety and depression. In our study, each participants' emotions were related to specific PICU-related sources of stress that changed over time and tended to be recurrent, as the child's health progressed and the PICU length of stay increased (Figure 3). Distressed families, especially in situations of potential end of life, need specific support to develop positive coping strategies (34). Our results show that appropriate support from experienced staff who know the child has a positive effect on families' emotional responses. This is supported by another study by Hagstrom where the PICU multidisciplinary team, especially nurses, were identified as key people to recognize families' needs early and provide them with emotional support. These competencies were crucial to help families navigate through the emotional roller coaster and potentially refer to specialist when required (6).

In our study, participants' experiences evolved from a state of shock to some kind of a “normal life” condition. Similarly, Geoghegan et al. showed that parents experience multiple phases of transition throughout a prolonged PICU hospitalization, which in turn result in an additional source of stress (10). Parents' concerns evolved from an acute to a “chronic” phase in which they were able to focus more on their own needs and that of the family. A phase of normalization in which they become familiar with the PICU environment and staff follows. Gaining familiarity allows parents to increase their knowledge acquisition and use their parental expertise (10). In our study, some participants struggle to be recognized as experts in their child's care by the PICU multidisciplinary team. This lack of recognition of parental role by HCPs has also been described in other studies causing unnecessary stress for families (8, 35, 36). We found in our study that it results in parental hypervigilance and/or conflictual relationships with HCPs. These results are in conflict with the fundamental values of family-centered care, where families should be considered as true partners in care of their child and fully empowered to be able to make informed decisions (28, 37). To achieve this, parents' individual needs of information and communication need to be considered (38), parents should be allowed to participate in their child's care as desired (39), and be informed and invited to participate in shared decision-making (40). Our study also highlight what caused the loss of a trusting relationship between participants and HCPs, and what facilitated it. Other studies support our findings and show that effective communication within the PICU multidisciplinary team influenced parents' involvement, and improves the ability of parents to affirm their needs and the competencies of HCPs to strengthen reciprocal trust (41–43). Bad news communication training proved to be useful for HCPs to tell parents that their child may not fully recover from his acute condition, but move to chronic or palliative care (43). More recently, Bedford and Bench advocated for an early intervention to support families, which starts at PICU admission and continues throughout the hospital stay. Although there is limited evidence, family education and follow-up interventions after PICU discharge have shown some benefits for the psychosocial health of families (44).

Care continuity emerged as a key issue for the participants, particularly when the need for individualized care was unmet. This issue has been poorly explored in the PICU literature. Nevertheless, two studies, exploring the needs of parents with complex medical conditions children, reported that provision of individualized care appears to be conditioned by three elementary abilities. The first one is the nurses' knowledge of their patients and family needs (7, 9). The second is the nurses' ability to coordinate patient care between the numerous members of the multidisciplinary team (45). The third is the capacity of the intensive care setting to provide comprehensive care (46). These studies show one perspective of individualized care, namely the nursing responsibilities to provide care that meet patients and family needs. Our findings, however, highlight the parental abilities to advocate for their child's needs and to promote individualized care for the child. In our study, some participants benefit from a primary care delivery model, in which a single senior nurse take the responsibility for the patient throughout their PICU stay, with varying degrees of success. Although not implemented for all, the benefits of such model has been reported and include decreased PICU length of stay (47), and improved parental involvement and child's quality of life (48).

Our study also highlights the common tendency of families to be together as much as possible, for instance by maintaining daily routines. A strategy to go back to their normality as much as possible; this was particularly predominant after a long PICU stay. This effort of cohesiveness seemed to give strength to the family unit and provide room to shift their attention from the sick child to the healthy siblings. Our results points out the negative impact of the child hospitalization on the siblings, but also their ability to develop some adaptation strategies. A recent review shows, however, the considerable variation in the adjustment and functioning of healthy siblings (49). Although research on siblings' experiences and long-term outcomes remains limited (50), it is an area that captured the attention of researchers (51). Our findings contribute to the body of knowledge in this relatively new area. Participants were able to highlight that opportunities to build family cohesiveness in including the siblings were limited because of the COVID-19 pandemic. The European Society of Pediatric and Neonatal Intensive Care (ESPNIC) produced patient and family-centered care recommendations, to facilitate families' participation in care and decision-making, and communication during family visits restrictions (52). The lack of family cohesion described by our participants during this period demonstrates that, despite the recommendations, PICU teams were unable to address the needs of the whole family. Perhaps this is due to a traditional model where care are centered on the parents rather than a more holistic view of what constitutes a family. Family nursing certainly offers an interesting avenue to guide HCPs to consider the families as a unit of care, identify families strengths and values (53), and offer family support interventions to mitigate the negative impact of the illness on the life and the relationships of all family members (54).

Because family nursing care considers the family as a system, it can be hypothesized that better parent physical, psychological and social health may be linked with better mental and physical health in children. The participants describe numerous coping strategies of varying degrees of adequacy. Family cohesiveness or the search for normality can be considered as adaptive coping strategies. The use of the language barrier, hypervigilance or distancing themselves from the attachment to the sick infant are maladaptive coping strategies. These results are in line with the literature, which highlights first that parents have to deal with their child's diagnosis and with the transformation of their parenthood, and consequently, parents implement coping strategies using PICU-related and non-related support (55). A meta-analysis showed that coping support interventions are effective to improve parents' anxiety and stress symptom burden. Included interventions were linked with parental education, emotional regulation and social support; it would be relevant to question the effect of these PICU-targeted interventions in families with CCI patients (56).

Participants underlined the uncertainties related to the future of their child and their family. However, for most of them, the uncertainties were balanced by a high feeling of hope that was present in their families. Hope is a positive emotion that has been reported by PICU parents and also identified as a coping mechanism by families (57). A recent study reports that, during and after discharge from PICU, parents consider functional, cognitive and emotional recovery, as well as their child's quality of life as the most important patient outcomes (58). Thus, evaluation and management of psychosocial outcomes of families in relation to the Post-Intensive Care Syndrome in Children (PICS-p) are now recommended, especially for families of CCI children (23, 59).

It was not intended to explore experience differences between mothers and fathers, but we were able to note that the stressors, needs and coping strategies of our mother and father participants were rather similar. Nevertheless, it appears that mothers were particularly affected by the non-recognition of their parental expertise, while fathers appeared to have high expectations regarding the administrative and financial support provided by the institution. This difference could be explained by the spontaneous repartition of roles between the two parents as the hospitalization progresses with a preponderant presence of mothers at the bedside, while fathers return to work or manage the household chores and family routine. The study by Jee et al. (60) also shows minimal differences in the needs, stressors and coping strategies between fathers and mothers. This later also shows that mothers spending longer periods at the bedside expressed higher needs of being informed and engaged, and higher stress to be away from their child, compared to fathers (60). Given the different types of family composition of current times, these results should be interpreted with caution as they may not reflect the experiences of single parent, blended families or rainbow families.

This study has several strengths and limitations. Strengths include the involvement of all Swiss PICUs, and quotations from different participants speaking three languages. In addition, transparency and trustworthiness of our findings are strongly supported. Several limitations should be noted. We did not seek feedback from participants on the research findings for ethical and logistical reasons. When this could have influenced the validity of the researchers' interpretation, this potential bias was minimized by using multiple coders and rigorous coding methods with explicit description of coding and memoring. Exclusion of families who did not speak or read French, German and English limited the cultural and ethnic diversity of the sample. Finally, family members were not included in all aspects of the design and delivery of the study, but they were consulted in the pre-test of the interview guide to ensure the questions were relevant and appropriate.

## Clinical Implications

The existing guidelines providing evidence-based strategies to optimize family-centered care in hospital and in the ICU are relevant for families of CCI patients (28, 37). Our study reveal a lack of implementation of those recommendations into practice, but also highlighted issues not addressed in these guidelines. Nevertheless, we propose the implementation of three main recommendations using an implementation framework to facilitate research uptake. First, family emotional support should be provided as early as possible from PICU admission, and tailored to the specific needs at the different critical time points of the PICU stay, including at PICU discharge (6, 53, 56). Second, care processes should be reviewed to provide true family-centered care in which parents are empowered (23, 44, 47, 48, 61). Third, individualized and coordinated care should be a priority to meet patient and family needs and provide the highest quality of care (38–43). Table 3 presents specific evidence-based recommendations that should address the practice gap highlighted in this study.

**Table 3:** Recommendations for the support of families with CCI patients.

**Family emotional support**
Provide early emotional family support, and refer to psychologist support when required (6, 28)
Integrate family nursing into standardized care, including systemic family assessment and intervention based on strengths of individual family members and of the family as a unit as well as on resources external to the family system (53)
Implement interventions to facilitate family coping: parental education, emotional regulation and social support (56)
**Family empowerment**
Ensure effective communication with families to establish a trusting relationship and facilitate involvement in care and empowerment (41–43). This can be achieved with routine interdisciplinary family conferences and ICU clinicians training in family-centered care communication (28). In times of pandemic, such as COVID-19, use of a decision-making tool is recommended (62)
Have an emphasis on the parents' individual needs regarding their desired level of information transmission, care participation, and shared decision-making (28, 38–40)
**Individualized coordinated care**
Implement primary and/or attending nurse (47, 61), and proactive palliative care consultations (28, 34, 48)
Prepare PICU discharge with families as early as possible and according to family individual needs (44)
Evaluation and management of family functioning after PICU discharge, including family quality of life, relationships, emotional function, and overall health (23, 59)

## Conclusions

Our study underlines the importance of caring for families with CCI children. Families report high negative emotional responses that affect their family functioning. Families experience is highly dependent on how HCPs are able to meet the parental needs, provide emotional support, reinforce parental empowerment, and allow high quality of care coordination. The implementation of interventions that truly meet the individual needs of families in this context is still to be improved.

## Data Availability Statement

The datasets generated for this study are available on request to the corresponding author.

## Ethics Statement

This study was approved by the Swiss Human Research Ethics Committees related to the eight sites with the leading committee being the CER-VD, project-ID: 2019-00944. Written informed consent to participate in this study was obtained for each participating family member.

## OCToPuS Consortium

Anne-Laure Lauria: Pediatric Intensive Care Unit, Department Woman-Mother-Child, Lausanne University Hospital, Lausanne, Switzerland; Angelo Polito and Nathalie Bochaton: Pediatric and Neonatal Intensive Care Unit, Department of Pediatrics, Gynecology and Obstetrics, University Hospitals of Geneva, Geneva, Switzerland; Daniel Trachsel: Pediatric Intensive Care and Pulmonology, University Children's Hospital Basel UKBB, Basel, Switzerland; Silvia Schnidrig and Tilman Humpl: Pediatric Intensive Care Unit, Department of Children and Adolescents, University Hospital, Bern, Switzerland; Bjarte Rogdo and Ellen Wild: Pediatric and Neonatal Intensive Care Unit, Children's Hospital of Eastern Switzerland, St. Gallen, Switzerland; Thomas Neuhaus and Sandra Stalder: Pediatric and Neonatal Intensive Care Unit, Department Children's Hospital, Lucerne Cantonal Hospital, Switzerland; Barbara Brotschi, Franziska von Arx and Anna-Barbara Schlüer: Department of Neonatal and Pediatric Intensive Care, University Children's Hospital, Zurich, Switzerland; Thomas Riedel and Pascale Van Kleef: Pediatric and Neonatal Intensive Care Unit, Department of Pediatrics, Cantonal Hospital Graubuenden, Chur, Switzerland.

## Author Contributions

CG, A-SR, M-HP, and M-CM contributed to conception and design of the study. CG and MM conducted the interviews. CG and PU performed the data analyses. CG wrote the first draft of the manuscript. A-SR wrote sections of the manuscript. All authors contributed to manuscript revision, read, and approved the submitted version.

## Funding

This study was funded by the Marisa Sophie Foundation Switzerland, the Anna & André Livio Glauser Foundation Switzerland, the Stiftung Pflegewissenschaft Schweiz, and an ESPNIC-Gettinge research grant.

## Conflict of Interest

The authors declare that the research was conducted in the absence of any commercial or financial relationships that could be construed as a potential conflict of interest.

## Publisher's Note

All claims expressed in this article are solely those of the authors and do not necessarily represent those of their affiliated organizations, or those of the publisher, the editors and the reviewers. Any product that may be evaluated in this article, or claim that may be made by its manufacturer, is not guaranteed or endorsed by the publisher.

## References

[1] Shapiro MC Henderson CM Hutton N Boss RD. Defining pediatric chronic critical illness for clinical care, research, and policy. Hosp Pediatr. (2017) 7:236–44. 10.1542/hpeds.2016-0107 28351944

[2] Polito A Combescure C Levy-Jamet Y Rimensberger P. Long-stay patients in pediatric intensive care unit: diagnostic-specific definition and predictors. PLoS ONE. (2019) 14:e0223369. 10.1371/journal.pone.0223369 31577836 PMC6774522

[3] Murphy Salem S Graham RJ. Chronic illness in pediatric critical care. Front Pediatr. (2021) 9:686206. 10.3389/fped.2021.686206 34055702 PMC8160444

[4] Marcus KL Henderson CM Boss RD. Chronic critical illness in infants and children: a speculative synthesis on adapting ICU care to meet the needs of long-stay patients. Pediatr Crit Care Med. (2016) 17:743–52. 10.1097/PCC.0000000000000792 27295581

[5] Alzawad Z Lewis FM Kantrowitz-Gordon I Howells AJ. A qualitative study of parents' experiences in the pediatric intensive care unit: riding a roller coaster. J Pediatr Nurs. (2020) 51:8–14. 10.1016/j.pedn.2019.11.015 31835065

[6] Hagstrom S. Family stress in pediatric critical care. J Pediatr Nurs. (2017) 32:32–40. 10.1016/j.pedn.2016.10.007 27884581

[7] Baird J Rehm RS Hinds PS Baggott C Davies B. Do you know my child? Continuity of nursing care in the pediatric intensive care unit. Nurs Res. (2016) 65:142–50. 10.1097/NNR.0000000000000135 26938363 PMC4780357

[8] de Man M Segers EW Schappin R van der Leeden K Wösten-van Asperen RM Breur H. Parental experiences of their infant's hospital admission undergoing cardiac surgery: a systematic review. Acta Paediatr. (2021) 110:1730–40. 10.1111/apa.15694 33251633 PMC8248104

[9] Rennick JE St-Sauveur I Knox AM Ruddy M. Exploring the experiences of parent caregivers of children with chronic medical complexity during pediatric intensive care unit hospitalization: an interpretive descriptive study. BMC Pediatr. (2019) 19:272. 10.1186/s12887-019-1634-0 31387555 PMC6683527

[10] Geoghegan S Oulton K Bull C Brierley J Peters M Wray J. The challenges of caring for long-stay patients in the PICU. Pediatr Crit Care Med. (2016) 17:e266–71. 10.1097/PCC.0000000000000725 27261667

[11] Geoghegan S Oulton K Bull C Brierley J Peters M Wray J. The experience of long-stay parents in the ICU: a qualitative study of parent and staff perspectives. Pediatr Crit Care Med. (2016) 17:e496–501. 10.1097/PCC.0000000000000949 27648895

[12] Wright-Sexton LA Compretta CE Blackshear C Henderson CM. Isolation in parents and providers of children with chronic critical illness. Pediatr Crit Care Med. (2020) e530–7. 10.1097/PCC.0000000000002344 32195899

[13] Markwalter DW Murphy MA Turnbull JM Fanning JB. Framing the future: family preparedness for care transitions of critically ill children. Fam Syst Health. (2019) 37:212–23. 10.1037/fsh0000431 31328928

[14] Bogetz JF Revette A DeCourcey DD. Clinical care strategies that support parents of children with complex chronic conditions. Pediatr Crit Care Med. (2021) 22:595–602. 10.1097/PCC.0000000000002726 33813549

[15] Zhang Y. Family functioning in the context of an adult family member with illness: a concept analysis. J Clin Nurs. (2018) 27:3205–24. 10.1111/jocn.14500 29700875 PMC6105391

[16] Leeman J Crandell JL Lee A Bai J Sandelowski M Knafl K. Family functioning and the well-being of children with chronic conditions: a meta-analysis. Res Nurs Health. (2016) 39:229–43. 10.1002/nur.21725 27128982

[17] Van Schoors M Caes L Knoble NB Goubert L Verhofstadt LL Alderfer MA. Systematic review: associations between family functioning and child adjustment after pediatric cancer diagnosis: a meta-analysis. J Pediatr Psychol. (2017) 42:6–18. 10.1093/jpepsy/jsw070 28173163

[18] Creswell JW Plano Clark VL. Designing and Conducting Mixed Methods Research. Los Angeles, CA: SAGE Publications (2014).

[19] Sandelowski M. Whatever happened to qualitative description? Res Nurs Health. (2000) 23:334–40. 10.1002/1098-240X(200008)23:4<334::AID-NUR9>3.0.CO;2-G 10940958

[20] Sandelowski M. What's in a name? Qualitative description revisited. Res Nurs Health. (2010) 33:77–84. 10.1002/nur.20362 20014004

[21] Creswell JW Poth CN. Philosophical assumptions and Interpretative Frameworks. In: Creswell JW, Poth CN, editors. Qualitative Inquiry and Research Design, Choosing Among Five Approaches. 4th ed. Thousand Oaks, CA: SAGE Publications. (2018). p. 53–91.

[22] Kelley AS Morrison RS Wenger NS Ettner SL Sarkisian CA. Determinants of treatment intensity for patients with serious illness: a new conceptual framework. J Palliat Med. (2010) 13:807–13. 10.1089/jpm.2010.0007 20636149 PMC4968277

[23] Manning JC Pinto NP Rennick JE Colville G Curley MAQ. Conceptualizing post intensive care syndrome in children-The PICS-p Framework. Pediatr Crit Care Med. (2018) 19:298–300. 10.1097/PCC.0000000000001476 29406379

[24] Carter MC Miles MS. The parental stressor scale: pediatric intensive care unit. Matern Child Nurs J. (1989) 18:187–98. 2491508

[25] Varni JW Sherman SA Burwinkle TM Dickinson PE Dixon P. The PedsQL family impact module: preliminary reliability and validity. Health Qual Life Outcomes. (2004) 2:55. 10.1186/1477-7525-2-55 15450120 PMC521692

[26] Latour JM Duivenvoorden HJ Tibboel D Hazelzet JA. The shortened EMpowerment of PArents in THe Intensive Care 30 questionnaire adequately measured parent satisfaction in pediatric intensive care units. J Clin Epidemiol. (2013) 66:1045–50. 10.1016/j.jclinepi.2013.02.010 23790723

[27] Chan KS Mangione-Smith R Burwinkle TM Rosen M Varni JW. The PedsQL: reliability and validity of the short-form generic core scales and Asthma Module. Med Care. (2005) 43:256–65. 10.1097/00005650-200503000-00008 15725982

[28] Davidson JE Aslakson RA Long AC Puntillo KA Kross EK Hart J. Guidelines for family-centered care in the neonatal, pediatric, and adult ICU. Crit Care Med. (2017) 45:103–28. 10.1097/CCM.0000000000002169 27984278

[29] Palinkas LA Horwitz SM Green CA Wisdom JP Duan N Hoagwood K. Purposeful sampling for qualitative data collection and analysis in mixed method implementation research. Adm Policy Ment Health. (2015) 42:533–44. 10.1007/s10488-013-0528-y 24193818 PMC4012002

[30] Mayring P. Qualitative Content Analysis: Theoretical Foundation, Basic Procedures and Software Solution Klagenfurt. (2014). Available from: http://nbn-resolving.de/urn:nbn:de:0168-ssoar-395173 (accessed on April 01, 2019).

[31] VERBI Software. MAXQDA 2020 [Computer Software]. Berlin: VERBI Software (2019).

[32] Kim H Sefcik JS Bradway C. Characteristics of qualitative descriptive studies: a systematic review. Res Nurs Health. (2017) 40:23–42. 10.1002/nur.21768 27686751 PMC5225027

[33] Saunders B Sim J Kingstone T Baker S Waterfield J Bartlam B. Saturation in qualitative research: exploring its conceptualization and operationalization. Qual Quant. (2018) 52:1893–907. 10.1007/s11135-017-0574-8 29937585 PMC5993836

[34] Zimmermann K Bergstraesser E Engberg S Ramelet AS Marfurt-Russenberger K Von der Weid N. When parents face the death of their child: a nationwide cross-sectional survey of parental perspectives on their child's end-of life care. BMC Palliat Care. (2016) 15:30. 10.1186/s12904-016-0098-3 26956995 PMC4784404

[35] Reid S Bredemeyer S Chiarella M. Integrative review of parents' perspectives of the nursing role in neonatal family-centered care. JOGNN. (2019) 48:408–17. 10.1016/j.jogn.2019.05.001 31150595

[36] Yeh J Ostini R. The impact of health literacy environment on patient stress: a systematic review. BMC Public Health. (2020) 20:749. 10.1186/s12889-020-08649-x 32448284 PMC7245697

[37] Institute for Patient and Family Centered Care. Advancing the Practice of Patient and Family Centered Care in Hospitals (2017). Available online at: https://www.ipfcc.org/resources/getting_started.pdf (accessed on May 30, 2019).

[38] Nelson JE Kinjo K Meier DE Ahmad K Morrison RS. When critical illness becomes chronic: informational needs of patients and families. J Crit Care. (2005) 20:79–89. 10.1016/j.jcrc.2004.11.003 16015521

[39] Uhm JY Choi MY. Mothers' needs regarding partnerships with nurses during care of infants with congenital heart defects in a paediatric cardiac intensive care unit. Inten Crit Care Nurs. (2019) 54:79–87. 10.1016/j.iccn.2019.07.003 31353190

[40] Wool J Irving SY Meghani SH Ulrich CM. Parental decision-making in the pediatric intensive care unit: an integrative review. J Fam Nurs. (2021) 27:154–67. 10.1177/1074840720975869 33523765

[41] Labrie NHM van Veenendaal NR Ludolph RA Ket JCF van der Schoor SRD van Kempen A. Effects of parent-provider communication during infant hospitalization in the NICU on parents: a systematic review with meta-synthesis and narrative synthesis. Patient Educ Couns. (2021) 104:1526–52. 10.1016/j.pec.2021.04.023 33994019

[42] Greenberg JA Gerhart J Horst JN Chen E Hunter RL O'Mahony S. A multidisciplinary team-based approach to improve communication with surrogates of patients with chronic critical illness patient and family centered actionable processes of care and performance measures for persistent and chronic critical ollness: a systematic review. Am J Hosp Palliat Care. (2020) 37:214–21. 10.1177/1049909119876606 31526015

[43] Boss RD Hirschfeld RS Barone S Johnson E Arnold RM. Pediatric chronic critical illness: training teams to address the communication challenges of patients with repeated and prolonged hospitalizations. J Pain Symptom Manage. (2020) 60:959–67. 10.1016/j.jpainsymman.2020.06.005 32540469

[44] Bedford ZC Bench S. A review of interventions supporting parent's psychological well-being after a child's intensive care unit discharge. Nurs Crit Care. (2019) 24:153–61. 10.1111/nicc.12405 30537005

[45] Graham RJ Pemstein DM Curley MA. Experiencing the pediatric intensive care unit: perspective from parents of children with severe antecedent disabilities. Crit Care Med. (2009) 37:2064–70. 10.1097/CCM.0b013e3181a00578 19384200

[46] Henderson CM Williams EP Shapiro MC Hahn E Wright-Sexton L Hutton N. “Stuck in the ICU”: caring for children with chronic critical illness. Pediatr Crit Care Med. (2017) 18:e561–e8. 10.1097/PCC.0000000000001332 28922265

[47] Edwards JD Jia H Baird JD. The impact of eligibility for primary attendings and nurses on PICU length of stay. J Crit Care. (2021) 62:145–50. 10.1016/j.jcrc.2020.12.006 33383307

[48] Boss R Nelson J Weissman D Campbell M Curtis R Frontera J. Integrating palliative care into the PICU: a report from the improving palliative care in the ICU advisory board. Pediatr Crit Care Med. (2014) 15:762–7. 10.1097/PCC.0000000000000209 25080152 PMC4184991

[49] Chin WL Jaaniste T Trethewie S. The role of resilience in the sibling experience of pediatric palliative care: what is the theory and evidence? Children (Basel). (2018) 5:97. 10.3390/children5070097 30012977 PMC6069058

[50] Abela KM Wardell D Rozmus C LoBiondo-Wood G. Impact of pediatric critical illness and injury on families: an updated systematic review. J Pediatr Nurs. (2020) 51:21–31. 10.1016/j.pedn.2019.10.013 31874458

[51] Manning JC Latour JM Curley MAQ Draper ES Jilani T Quinlan PR. Study protocol for a multicentre longitudinal mixed methods study to explore the Outcomes of ChildrEn and fAmilies in the first year after paediatric Intensive Care: the OCEANIC study. BMJ Open. (2020) 10:e038974. 10.1136/bmjopen-2020-038974 32423943 PMC7239532

[52] Rimensberger PC Kneyber MCJ Deep A Bansal M Hoskote A Javouhey E. Caring for critically ill children with suspected or proven Coronavirus disease 2019 infection: recommendations by the scientific sections' collaborative of the European Society of Pediatric and Neonatal Intensive Care. Pediatr Crit Care Med. (2021) 22:56–67. 10.1097/PCC.0000000000002599 33003177 PMC7787185

[53] International Family Nursing Association. IFNA Position Statement on Advanced Practice Competencies for Family Nursing. (2017). Available online at: https://internationalfamilynursing.org/2017/05/19/advanced-practice-competencies/ (accessed on June 15, 2021).

[54] Bell JM. Family nursing is more than family centered care. J Fam Nurs. (2013) 19:411–7. 10.1177/1074840713512750 24227014

[55] Jackson AC Higgins RO Frydenberg E Liang RP Murphy BM. Parent's perspectives on how they cope with the impact on their family of a child with heart disease. J Pediatr Nurs. (2018) 40:e9–e17. 10.1016/j.pedn.2018.01.020 29396310

[56] Doupnik SK Hill D Palakshappa D Worsley D Bae H Shaik A. Parent coping support interventions during acute pediatric hospitalizations: a meta-analysis. Pediatrics. (2017) 140:171. 10.1542/peds.2016-4171 28818837 PMC5574731

[57] Cabeça LPF Melo LL. From despair to hope: copying of relatives of hospitalized children before bad news report. Rev Bras Enferm. (2020) 73:e20200340. 10.1590/0034-7167-2020-0340 33206910

[58] Fayed N Cameron S Fraser D Cameron JI Al-Harbi S Simpson R. Priority outcomes in critically ill children: a patient and parent perspective. Am J Crit Care. (2020) 29:e94–e103. 10.4037/ajcc2020188 32869071

[59] Fink EL Maddux AB Pinto N Sorenson S Notterman D Dean JM. A core outcome set for pediatric critical care. Crit Care Med. (2020) 48:1819–28. 10.1097/CCM.0000000000004660 33048905 PMC7785252

[60] Jee RA Shepherd JR Boyles CE Marsh MJ Thomas PW Ross OC. Evaluation and comparison of parental needs, stressors, and coping strategies in a pediatric intensive care unit. Pediatr Crit Care Med. (2012) 13:e166–72. 10.1097/PCC.0b013e31823893ad 22079953

[61] Siow E Wypij D Berry P Hickey P Curley MA. The effect of continuity in nursing care on patient outcomes in the pediatric intensive care unit. J Nurs Adm. (2013) 43:394–402. 10.1097/NNA.0b013e31829d61e5 23892304

[62] Institute for Patient and Family Centered Care. Family Presence During a Pandemic: Guidance for Decision-Making. Available online at: https://www.ipfcc.org/bestpractices/covid-19/IPFCC_Family_Presence.pdf (accessed on June 15, 2021).

